# Catalytic Hydrogenation of Phenolic Compounds Using Transition Metal Oxides Deposited on a Carbon Sorbent from Coke Fines

**DOI:** 10.3390/molecules31091455

**Published:** 2026-04-28

**Authors:** Aigul T. Ordabaeva, Zainulla M. Muldakhmetov, Mazhit G. Meiramov, Sergey V. Kim

**Affiliations:** Institute of Organic Synthesis and Chemistry of Coal of Kazakhstan Republic, Alikhanov Str., 1, Karaganda 100012, Kazakhstan

**Keywords:** catalysts based on transition metals, cobalt oxide, carbon sorbent, coke fines, hydrogenation of phenols

## Abstract

The purpose of this work was to synthesize and study catalytic systems based on a carbon-containing support obtained from coke fines from the Shubarkol deposit as a waste product of the coal industry for the processing of phenolic compounds. Based on the obtained carbon sorbent, mono- and binary catalysts with active phases of transition metal oxides (Fe, Co, Ni) were synthesized by wet impregnation, followed by heat treatment at 500–700 °C, as well as the aluminum oxide compositions. The surface morphology and elemental composition of the samples were studied by scanning electron microscopy (SEM) with energy dispersion analysis and elemental mapping (EDS mapping), and the content of active phases was determined using inductively coupled plasma optical emission spectrometry (ICP-OES). The catalytic activity was studied in phenol hydrogenation reactions. The CoO/C catalyst demonstrated the greatest activity, providing a 62.36% benzene yield during phenol hydrogenation. The catalytic activity of the CoO/C catalyst has also been studied in the hydrogenation reactions of structurally and functionally more complex compounds, pyrocatechol and resorcinol. The yield of benzene was 63.16% in the hydrogenation of pyrocatechol and 48.64% in the hydrogenation of resorcinol. It was found that the CoO/C catalyst exhibits the highest efficiency at a temperature of 420 °C, a pressure of 6–6.5 MPa and a reaction duration of 120 min. The results obtained make it possible to evaluate the prospects of using a carbon sorbent obtained from coke fines from the Shubarkol deposit as a support for CoO as part of an active and stable catalytic system designed for deep processing of phenolic compounds.

## 1. Introduction

Phenolic compounds are widely distributed in industrial wastewater, coal and biomass pyrolysis products, as well as in spent petroleum products [1]. Despite their value as a chemical raw material, the presence of phenols in waste poses a serious environmental threat due to their toxicity, resistance to biodegradation, and negative effects on living organisms [2].

The catalytic conversion of phenolic compounds, including phenol and its alkyl—substituted derivatives, into aromatic hydrocarbons (benzene, toluene, xylenes) is a promising area of utilization that provides not only a reduction in environmental stress, but also the production of high—demand raw materials for the petrochemical and polymer industries [3,4].

Catalytic hydrogenation and hydrodeoxygenation (HDO) of phenolic substrates make it possible to selectively break C–O bonds and reduce the oxygen content in products to a level acceptable for fuel and chemical applications [5,6].

In recent years, a significant body of work has been published on the hydrogenation of phenol and model lignin derivatives of phenols on various metal catalysts. Most studies in the field of catalytic conversion of phenols are devoted to systems based on noble metals (Pd, Pt, Ru), which under mild conditions provide high activity and selectivity: depending on the reaction conditions, they are able to direct the conversion of phenol either into saturated products (cyclohexanol, cyclohexanone), or—at elevated temperatures—into benzene through dehydroxylation [5,7]. However, the high cost and limited availability of precious metals stimulate the search for alternative catalysts based on inexpensive transition metals (Ni, Co, Fe). Such systems have already demonstrated promising results in hydrodeoxygenation reactions of phenolic substrates obtained from lignin and other types of biomass [8,9,10].

Heterogeneous catalysts capable of effectively removing hydroxyl groups from the aromatic ring (dehydroxylation) play a key role in such processes without saturating it [3,11].

The process of hydrodeoxygenation (HDO) of guaiacol on a Ni–Fe catalyst deposited on carbon nanotubes (CNT) was studied [12]. At a temperature of 250 °C and a pressure of 2 MPa of hydrogen, 95.8% conversion of guaiacol was achieved to form benzene with a selectivity of 88.3%. It is important that under these conditions, the hydroxyl group was selectively removed without hydrogenation of the aromatic ring, which indicates the dominance of the dehydroxylation (DDO) pathway over hydrogenation. This demonstrates the high efficiency of the Ni–Fe/CNT catalyst in preserving the aromatic structure of the products [12].

Studies show that Ni–Fe catalysts selectively destroy C–O bonds in phenolic compounds, preserving the aromatic structure of the molecule [13]. At a temperature of 350 °C and a pressure of 2 MPa H_2_, the Ni_3_Fe/SiO_2_ catalyst provided an almost complete yield of benzene (98.5%) from phenol at almost 100% conversion, which indicates a high selectivity of dehydroxylation. The high efficiency is explained by the synergy between the Ni–Fe metal centers and the oxygen groups of the support, which ensures the activation of C–O bonds without saturating the aromatic ring [13].

CoO-based catalysts exhibit high activity in hydrogenation and hydrodeoxygenation reactions of phenolic compounds.

It was also found that a heterogeneous catalyst based on CoO nanoparticles immobilized on porous carbon (CoO_x_@CN) provides chemoselective hydrogenation of phenol to cyclohexanol with almost quantitative yield (98%) and selectivity above 99% at 150 °C and pressure H_2_ 3 MPa in an aqueous medium [14]. The high efficiency of the catalyst is due to the synergy between two components: Co_3_O_4_, which promotes the adsorption and activation of the phenolic ring due to the interaction of π electrons with positively charged oxide centers, and metallic Co, formed in situ during the reaction and responsible for the dissociation of molecular hydrogen. The homogeneous dispersion of the nanoparticles and the high specific surface area of the support further enhance the catalytic activity. Thus, the combination of structural and functional features of CoO_x_@CN makes it possible to implement an efficient, inexpensive and environmentally preferable process for the production of cyclohexanol, a key intermediate for the synthesis of nylon-6 [14].

Sulfidated CoMo catalysts deposited on mixed Al_2_O_3_–TiO_2_ oxide (Al/Ti = 2, designated as AT2) exhibit significantly increased activity in the hydrodeoxygenation (HDO) of phenol compared with traditional CoMo/Al_2_O_3_ [15]. At a temperature of 593 K and an H_2_ pressure of 5.4 MPa, the CoMo/AT2 catalyst demonstrated an 85% higher initial reaction rate, providing predominantly direct dehydroxylation (DDO pathway) to form benzene with almost complete suppression of aromatic ring hydrogenation. The high efficiency is explained by the weakened interaction of the metal with the support, which contributes to the formation of easily reducible oxide precursors of Co and Mo in octahedral coordination and, as a result, an increase in the proportion of the active phase of CoMoS due to the better availability of the cobalt promoter for decorating the edges of MoS_2_ layers. In addition, the CoMo/AT2 catalyst showed increased resistance to CS_2_ inhibition: when 200 ppm of sulfur was added, the decrease in activity was only 52% versus 69% for CoMo/Al_2_O_3_, which indicates a lower sensitivity of the DDO route to blocking S vacancies at the promoted edges of MoS_2_ [15].

At the same time, the choice of support has a significant effect on the dispersion of the active phase, its resistance to sintering and, as a result, on the catalytic efficiency of the system [16].

Traditionally, Al_2_O_3_, SiO_2_, zeolites or nanomaterials are used as supports [17,18,19].

However, in recent years, carbon materials obtained from biomass have been actively studied due to their high porosity, chemical inertia, and developed surface [20,21,22].

Nevertheless, data on the use of carbon sorbents based on coke fines as supports for phenol dehydroxylation catalysts remain extremely limited, especially in comparison with traditional supports.

In this regard, the purpose of this work is to synthesize a series of composite catalysts based on transition metal oxides (Fe, Co, Ni) deposited on a carbon sorbent obtained in a previous study by activating coke fines from the Shubarkol deposit [23] and Al_2_O_3_, as well as to study their physicochemical characteristics and catalytic activity in phenol hydrogenation reactions. The following catalysts were obtained by impregnating supports in aqueous solutions of transition metal salts: Fe_2_O_3_/C; CoO/C; NiO/C; CoO/Al_2_O_3_; Fe_2_O_3_–CoO/C; Fe_2_O_3_–NiO/Al_2_O_3_ and NiO–CoO/Al_2_O_3_

The quantitative composition and morphology of the surface of the carbon sorbent and the resulting catalysts were determined using scanning electron microscopy and energy dispersion spectroscopy with elemental mapping (EDS mapping). Inductively coupled plasma optical emission spectrometry (ICP-OES) was used to perform quantitative elemental analysis of the obtained catalysts, which are metal oxides of the iron subgroup (Fe, Co, Ni) deposited on carbon and aluminum oxide-based supports. The catalytic activity of the synthesized catalysts in the hydrogenation of phenolic compounds has been established.

The yield of hydrogenation products was determined using gas–liquid chromatography (GLC).

**Figure 1:**
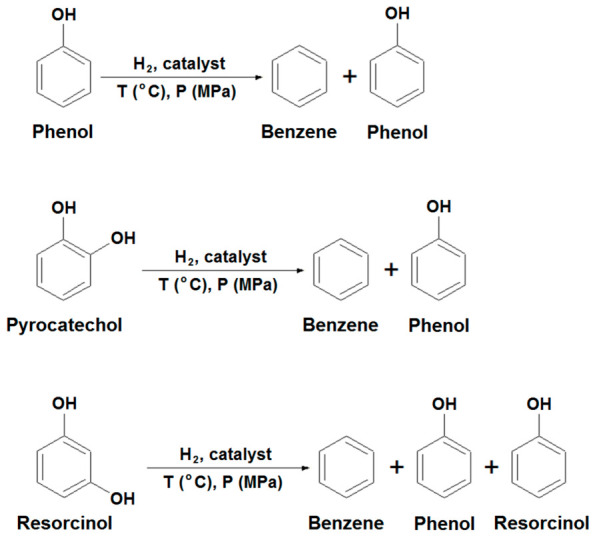
General view of the scheme of hydrogenation of phenol, pyrocatechol and resorcinol.

A general view of the hydrogenation scheme of phenol, pyrocatechol, and resorcinol is shown in Figure 1.

## 2. Results

**Figure 2:**
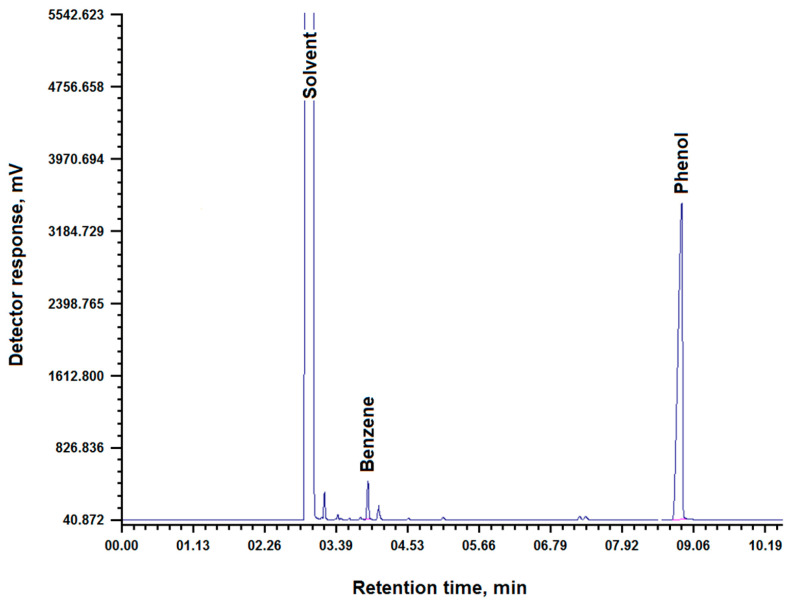
Chromatogram of phenol hydrogenation in the presence of the resulting sorbent carbon.

To evaluate the intrinsic catalytic activity of the carbon carrier used, obtained in previous studies [23], and to isolate the contribution of cobalt to the process of phenol hydrogenation, an experiment was conducted on phenol hydrogenation in the presence of a carbon sorbent without deposited metal. The chromatogram of the reaction products and the quantitative composition of the reaction mixture are shown in Table 1 and Figure 2.

The data obtained in Table 1 and Figure 2 demonstrate an insignificant conversion of phenol.

The catalytic activity of the synthesized catalysts was studied in the reaction of phenol hydrogenation. The following catalysts were obtained by impregnating supports in aqueous solutions of transition metal salts: Fe_2_O_3_/C; CoO/C; NiO/C; CoO/Al_2_O_3_; Fe_2_O_3_–CoO/C;Fe_2_O_3_–NiO/Al_2_O_3_ and NiO–CoO/Al_2_O_3_

The results of the analysis of synthesized catalysts by inductively coupled plasma optical emission spectrometry (ICP-OES) are presented in Table 2.

As a result of the study, a quantitative elemental analysis of five catalyst samples representing metal oxides of the iron subgroup (Fe, Co, Ni) deposited on carbon and aluminum oxide supports was performed using inductively coupled plasma optical emission spectrometry (ICP-OES). The data obtained made it possible to evaluate the effectiveness of implantation of active components and the purity of the matrix.

The aluminum content varies from 1.144 to 1.404% in the first four samples (No. 1–4), which indicates the presence of insignificant amounts of additives contained in the support. The content of more than 50% was recorded in sample No. 5 (Fe_2_O_3_–NiO/Al_2_O_3_), which clearly indicates the use of aluminum oxide as the main support.

The maximum iron content is observed in sample No. 1 Fe_2_O_3_/C (8.598%), which corresponds to its composition. In samples with mixed active phases of Fe-Co and Fe-Ni, the iron content is 4.380 and 3.273%, respectively, which confirms the partial replacement or co-incorporation of other metals. The CoO/C and NiO/C samples show only trace amounts of iron (<1%), which indicates a high selectivity of synthesis.

The expressed Co content is recorded in CoO/C (4.4119%) and especially in Fe_2_O_3_–CoO/C (4.8349%), which confirms the successful introduction of Co into the support. Other samples contain only small amounts of Co (<0.05%), which may be due to cross-contamination.

A high nickel content was found in the NiO/C samples. (6.1210%) and Fe_2_O_3_–NiO/Al_2_O_3_(5.4620%), which confirms the effective deposition of nickel components on the support. In the remaining samples, the nickel level is at the background level (<0.02%).

The chromium content in all samples is <0.01%, which indicates trace amounts of impurities in the starting materials, which may be due to contamination transferred from the equipment during sample preparation. The highest value (0.0125%) was recorded in the Fe_2_O_3_–CoO/C sample. The data obtained made it possible to evaluate the effectiveness of implantation of active components and the purity of the matrix.

The catalytic activity of the synthesized catalysts was studied in the reaction of phenol hydrogenation.

The catalytic activity was evaluated based on the yield of benzene. The results are presented in Table 3.

**Figure 3:**
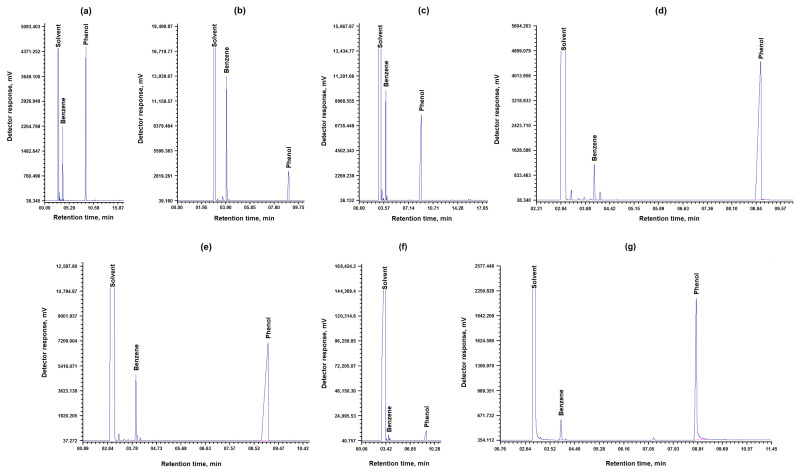
Chromatogram of phenol hydrogenation in the presence of the following catalysts: (**a**) Fe_2_O_3_/C; (**b**) CoO/C; (**c**) NiO/C; (**d**) CoO/Al_2_O_3_; (**e**) Fe_2_O_3_–CoO/C; (**f**) Fe_2_O_3_–NiO/Al_2_O_3_; (**g**) NiO–CoO/Al_2_O_3_.

Chromatograms of phenol hydrogenation in the presence of the obtained Fe_2_O_3_/C, CoO/C, NiO/C, CoO/Al_2_O_3_, Fe_2_O_3_–CoO/C, Fe_2_O_3_–NiO/Al_2_O_3_ and NiO–CoO/Al_2_O_3_ catalysts are shown in Figure 3.

It was found that the highest yield of benzene is observed when using a CoO/C catalyst at 420 °C, a hydrogen pressure of 6.5 MPa and a process duration of 120 min.

An analysis of the results from Table 1 and the chromatograms in Figure 1 showed that CoO/C exhibits significantly higher activity than the rest of the systems, with a catalytic activity of 62.36% in benzene yield, which is 3–6 times higher than that of the other systems studied. This indicates the high efficiency of CoO in combination with a carbon support obtained from coke fines for the dehydroxylation reaction.

Carbon-based monoxide catalysts (Fe_2_O_3_/C, NiO/C) exhibit low activity, despite the presence of a significant amount of metal (according to ICP-OES data: Fe—8.60%, Ni—6.12%). This indicates that the mere presence of metal is not enough—a specific interaction with the support or a certain surface condition is required (for example, the degree of reduction, phase dispersion) [24].

Binary catalysts (Fe_2_O_3_–CoO/C, Fe_2_O_3_–NiO/Al_2_O_3_ and NiO–CoO/Al_2_O_3_) demonstrated lower catalytic activity compared not only with CoO/C, but also with NiO/C. This may be due to the lack of a synergistic effect between the components in the selected formulations, partial blocking of the active centers of Co or Ni by Fe, as well as an unfavorable interaction with the support, in particular, the strong binding of nickel to Al_2_O_3_, which reduces its availability for the catalytic reaction.

Low activity of the Fe_2_O_3_–NiO/Al_2_O_3_ system (12.53% benzene) and the NiO–CoO/Al_2_O_3_ system (9.65% benzene), compared to the corresponding carbon-supported mono-oxide catalysts (Fe_2_O_3_/C—10.53%; NiO/C—18.17%) may be attributed to the strong interaction of nickel with aluminum oxide, which limits the availability of active sites. This is consistent with the literature data indicating that carbon supports, due to their chemical inertia and weak metal–support interaction, contribute to better preservation of reactive transition metal centers [13,24].

**Figure 4:**
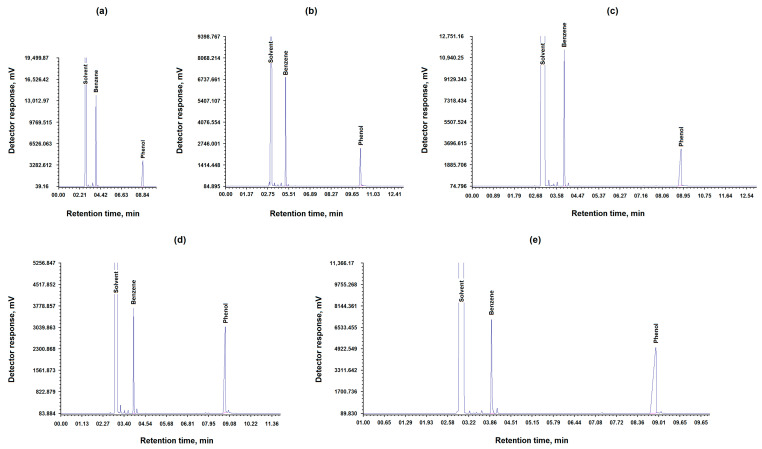
Chromatograms of phenol hydrogenation in the presence of the obtained CoO/C catalyst during 5 cycles (**a**–**e**).

It was found that the CoO/C catalyst is most effective at a temperature of 420 °C, a pressure of 6–6.5 MPa, and a reaction time of 120 min, and retains its catalytic activity for five cycles. The results are presented in Table 4 and Figure 4.

Table 4 shows that the activity of the CoO/C catalyst decreases by almost half after the third cycle.

**Figure 5:**
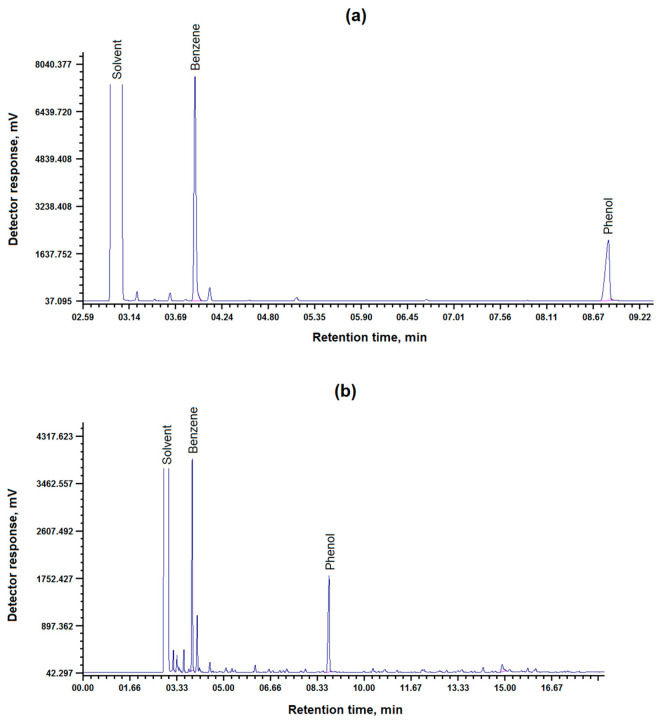
Chromatogram of the hydrogenation products of (**a**) pyrocatechol and (**b**) resorcinol in the presence of a CoO/C catalyst.

The catalytic activity of the CoO/C catalyst has also been studied in the hydrogenation reactions of structurally and functionally more complex compounds, pyrocatechol and resorcinol. The chromatograms are shown in Figure 5 and in Table 5.

Pyrocatechol (orthodiol) is converted to benzene more efficiently than phenol (63.16% versus 62.36%). The higher yield of benzene from pyrocatechol compared with phenol may be due to the electronic and structural influence of the second hydroxyl group in the ortho position, which helps to weaken the C–O bonds and stabilize the transition state on the catalyst surface [25].

It was found that hydrogenation of pyrocatechol in the presence of the synthesized CoO/C catalyst, the highest yield of benzene 63.16% and phenol 36.84% is achieved at a temperature of 420 °C, a process duration of 120 min and a hydrogen pressure of 6.5 MPa.

Hydrogenation of resorcinol using the obtained CoO/C catalyst showed that the highest yield of benzene 48.64% and phenol 45.82% is achieved at a temperature of 420 °C, a process duration of 120 min and a hydrogen pressure of 6 MPa.

The use of the CoO/C catalyst in the hydrogenation of resorcinol (metadiol) demonstrates a noticeably lower yield of benzene (48.64%) than in the hydrogenation of pyrocatechol (63.16%). This may be due to steric and electronic difficulties in breaking the second C–O bond in the meta position. In particular, the presence of a second hydroxyl group can stabilize the aromatic ring due to additional electron delocalization, making dehydroxylation more difficult, which is consistent with the reduced reactivity of resorcinol in comparison with other dihydroxybenzene isomers [26,27]. Competitive adsorption inhibition is also possible: simultaneous fixation of two –OH groups on the surface reduces the mobility of the molecule and slows down dehydroxylation [28]. The structure of the carbon support is a critical factor determining the dispersion of metal nanoparticles and their resistance to sintering in hydrodeoxygenation (HDO) reactions. The properties of the carrier, including its surface chemistry and morphology, directly affect the metal–carrier interaction, which makes it possible to fix the active centers and prevent their deactivation under harsh reaction conditions [8]. For carbon materials, it has been shown that optimal carrier properties contribute to high metal dispersion and improve catalytic performance, while regulating the balance between graphite and amorphous domains increases the number of available active sites and ensures their stability during the conversion of lignin-derived phenolics [8].

In heterogeneous catalysis, a similar structural balance can potentially contribute to better dispersion and stability of the active metal phase, although this issue requires separate experimental confirmation.

The absence of other products in Table 2 (cyclohexanone, cyclohexanol, dimers, etc.) suggests a high selectivity of CoO/C to the aromatic pathway (dehydroxylation without saturation of the ring), which is a favorable result for obtaining benzene as the target product. The absence of saturation products (cyclohexanol, cyclohexane) in our experiments indicates the dominance of the dehydroxylation (DDO) pathway over the hydrogenation of the aromatic ring. This behavior is consistent with the results of Lonchay et al., where Fe/ZrO_2_ also demonstrated selective removal of hydroxyl groups while preserving the aromatic structure, which was associated with the oxyphilicity of the metal phase and moderate acidity of the support [29].

**Figure 6:**
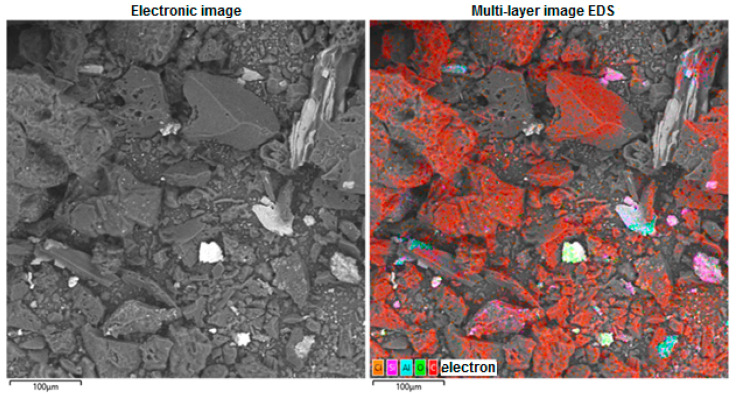
Scanning electron microscopy and energy dispersion spectroscopy with elemental mapping (EDS mapping) of the resulting carbon sorbent, which is used as a catalyst substrate.

The analysis of scanning electron microscopy and energy dispersion spectroscopy with elemental mapping (EDS mapping) of the obtained carbon sorbent used as a substrate of the CoO/C catalyst is shown in Figure 6.

**Figure 7:**
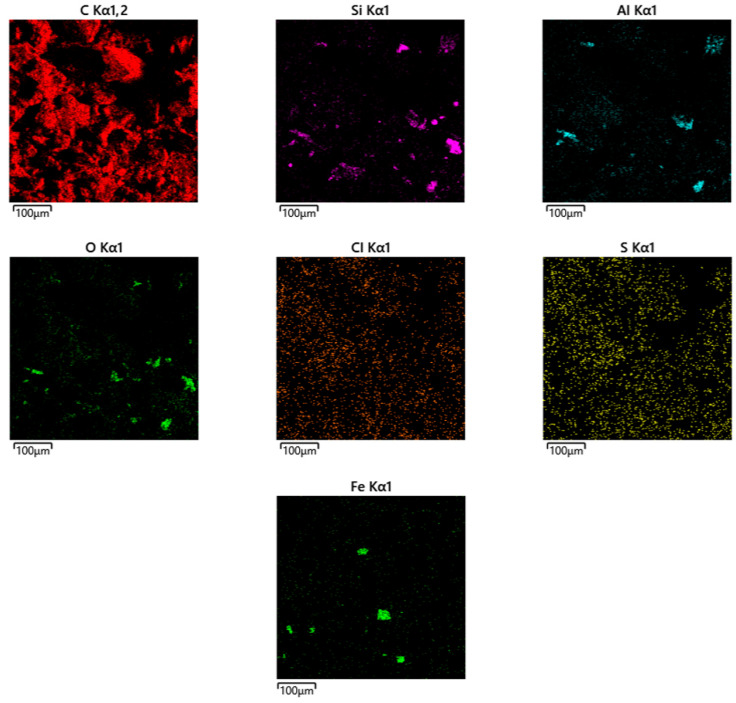
Element-by-element mapping using energy dispersive X-ray spectroscopy (EDS/EDX mapping) for the resulting carbon sorbent, which is used as a catalyst substrate.

Piecemeal mapping using energy dispersive X-ray spectroscopy (EDS/EDX mapping) for the resulting carbon sorbent, which is used as a substrate for the CoO/C catalyst, is shown in Figure 7.

**Figure 8:**
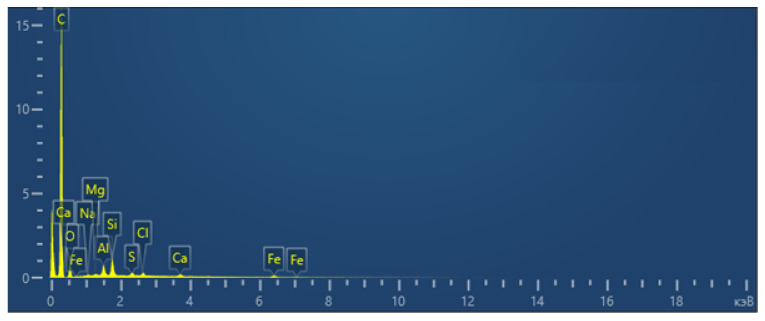
The total spectrum of the element content map in the resulting carbon sorbent, which is used as a catalyst substrate.

The total spectrum of the element content map in the resulting carbon sorbent, which is used as a catalyst substrate, is shown in Figure 8.

The interpretation of the total spectrum of the element content map in the resulting carbon sorbent, which is used as a catalyst substrate, is presented in Table 6.

As a result of studies conducted using scanning electron microscopy and energy dispersion spectroscopy with elemental mapping (EDS mapping), it was found that the main element in the obtained carbon sorbents is carbon (C). Its high content (>86% by weight and >91% by atomic fraction) indicates the organic or carbonaceous nature of the material. A significant proportion of oxygen (8.36%) indicates the presence of oxides and functional groups (hydroxyls, carboxyls, etc.). Trace amounts of the elements Na, Mg, Al, Si, S, Cl, Ca, and Fe may indicate impurities, residues of inorganic phases and the presence of particles or inclusions, possibly of mineral origin. The presence of Si, Al, Ca, and Fe may indicate the presence of mineral particles, such as clay, aluminum oxides, silicates, or fragments of earth dust. The analysis of the distribution of chemical elements on the sample surface was carried out using energy dispersion spectroscopy with element mapping (EDS mapping) on a scanning electron microscope.

### CoO/C Catalyst

**Figure 9:**
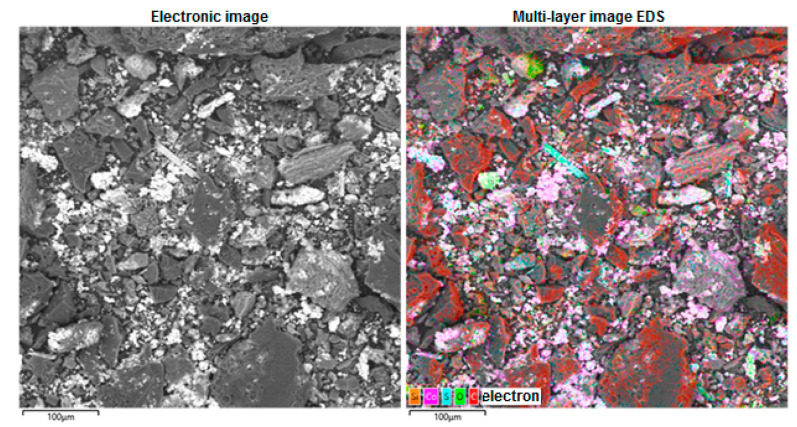
Scanning electron microscopy and energy dispersion spectroscopy with elemental mapping (EDS mapping) of the obtained CoO/C catalyst.

Scanning electron microscopy and energy dispersion spectroscopy with elemental mapping (EDS mapping) of the obtained CoO/C catalyst are shown in Figure 9.

**Figure 10:**
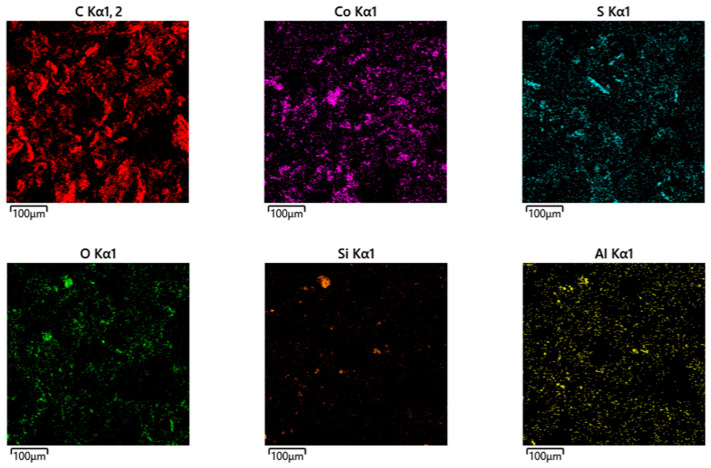
Element-by-element mapping using energy dispersive X-ray spectroscopy (EDS/EDX mapping) for the obtained CoO/C catalyst.

Element-by-element mapping using energy dispersive X-ray spectroscopy (EDS/EDX mapping) for the resulting CoO/C catalyst is shown in Figure 10.

**Figure 11:**
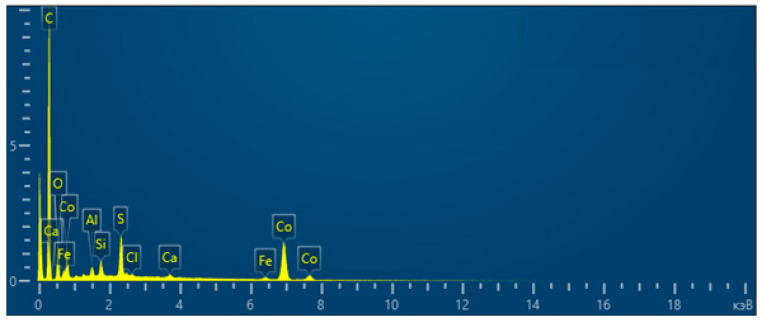
The total spectrum of the map of the element content in the obtained CoO/C catalyst.

The total spectrum of the element content map in the obtained CoO/C catalyst is shown in Figure 11.

The interpretation of the total spectrum of the element content map in the obtained CoO/C catalyst is presented in Table 7.

As a result, using scanning electron microscopy and energy dispersion spectroscopy with elemental mapping (EDS mapping), it was found that the CoO/C catalyst contains a significant amount of carbon (67.75%), which indicates the presence of a carbon support or matrix. The significant content of Co—19.96% by weight and 5.14% by atomic fraction—confirms the presence of an active phase. At the same time, the ratio of Co:O involves the formation of oxide compounds such as CoO or Co_3_O_4_. The presence of oxygen (7.57%) additionally confirms the oxide nature of the Co phase. Detected sulfur (2.34%) may be a residue of sulfur-containing precursors or synthesis products. The presence of Fe, Al, Si, Ca, and Cl may be related to the residual components of the mineral part.

**Figure 12:**
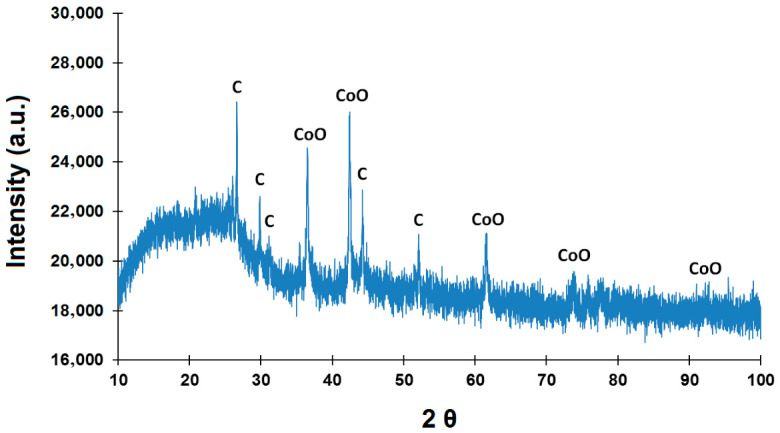
Diffractogram of the CoO/C catalyst.

The results of the CoO/C X-ray phase analysis are shown in Figure 12.

According to the X-ray phase analysis data shown in Figure 12, it can be seen that the CoO/C sample is a composite system consisting of three phases:

- CoO, crystallizing in a cubic structure of the NaCl type (spatial group (Fm3 m) with lattice parameters *a* = *b* = 4.2627(15) Å, *c* = 4.265(2) Å;
- graphite-2H, characterized by a hexagonal structure (P6_3_/mmc) with *a* = *b* = 2.4817(9) Å, *c* = 6.693(4) Å;
- an additional graphite-like phase, which can be attributed to a partially ordered carbon material with orthorhombic symmetry (*a* = 4.94(2) Å, *b* = 5.986(5) Å, *c* = 4.361(7) Å).

The most intense CoO reflexes are observed at 2θ = 36.47° (111), 42.37° (200) and 61.48° (220). The next peak in intensity was recorded at 73.64° (311); despite the fact that the reflex (222) is theoretically expected at 77.49°, it is less pronounced on the experimental diffractogram than (311), possibly due to microstress in the crystal lattice and an increase in the background signal in the region of high angles 2θ.

The functional properties of carbon materials may depend on the balance between partially graphitized and amorphous domains. For example, studies of anodes for potassium-ion batteries have shown that the combination of ordered graphite regions (providing conductivity and interlayer channels) with an amorphous phase (improving kinetics and buffering volumetric changes) enhances the efficiency of the carbon matrix [29]. In heterogeneous catalysis, a similar structural balance can potentially contribute to better dispersion and stability of the active metal phase, although this issue requires separate experimental confirmation.

## 3. Discussion

The results obtained demonstrate that the CoO/C catalyst synthesized on a carbon sorbent obtained from coke fines from the Shubarkol deposit has a high catalytic activity in the reactions of dehydroxylation of phenolic compounds to form benzene. The yield of the target product, benzene—reaches 62.36% when phenol is hydrogenated, 63.16% when pyrocatechol is converted, and 48.64% when resorcinol is used as a substrate. These data significantly exceed the indicators of other studied systems based on Fe, Ni and their binary compositions, which emphasizes the predominant catalytic role of cobalt oxide in combination with a carbon support. Under these conditions, the CoO/C catalyst demonstrates a significantly higher yield of benzene compared to systems based on Fe, Ni and their binary compositions.

The superiority of CoO/C over mono- and binary analogs (Fe_2_O_3_/C, NiO/C, CoO/Al_2_O_3_, Fe_2_O_3_–CoO/C, Fe_2_O_3_–NiO/Al_2_O_3_ and NiO–CoO/Al_2_O_3_) can be explained by several factors. Firstly, the carbon support obtained from coke fines is characterized by a high carbon content (>86%) and, based on the nature of carbon materials of this type, can provide a developed surface for dispersing the active phase.

Bimetallic catalysts have a significant decrease in activity, possibly due to the interactions of active phase metals with each other and with the substrate material, leading to a decrease in the reactivity of the catalyst. The decrease in system activity can be explained by the nature of the support. As shown in [30], Al_2_O_3_, having an acidic surface, promotes strong interaction with transition metals, which leads to partial “fixation” of the active phase and its reduced availability. At the same time, carbon supports, being neutral and inert, provide weak metal–support interaction, high dispersion, and resistance to inhibition, which is consistent with the high activity and selectivity of CoO/C in phenolic dehydroxylation [24].

The choice of a carbon support for Co-based catalysts is due to the need to weaken the metal–support interaction (MSI). According to a modern review of the literature [31,32], carbon materials are characterized by a weaker interaction with Co compared to traditional oxide supports (Al_2_O_3_, SiO_2_, TiO_2_). Unlike oxides, where strong MSI leads to the formation of inactive mixed oxides (for example, spinel CoAl_2_O_4_), carbon supports promote easier reduction in the active phase of Co and prevent the formation of compounds that are difficult to reduce.

A comparative description of the optimal conditions for the catalytic treatment of phenols and benzene yields using the catalysts obtained in this work and in the literature data is presented in Table 8.

The combination of XRD data (Figure 12) and synthesis conditions indicates the dominance of the crystalline phase of CoO. Nevertheless, we recognize that the XRD method does not completely exclude the presence of trace amounts of highly dispersed or amorphous Co_3_O_4_, whose reflexes may be blurred or below the detection threshold.

It is possible that CoO with Co in the oxidation state +II in the resulting catalyst has sufficient oxyphilicity to activate the C–O bond in phenolic compounds without hydrogenation of the aromatic ring. This creates favorable conditions for the reaction to proceed by the mechanism of direct dehydroxylation (DDO) to form benzene.

In contrast, Co_3_O_4_, which is mixed-valent Co(II)/Co(III), is characterized by a higher tendency to activate the π-system of the aromatic ring and to dissociate hydrogen, especially under reducing conditions with partial reduction to elemental Co(0) [14]. This may promote hydrogenation pathways leading to the formation of cyclohexanol and cyclohexane.

Thus, the dominance of the CoO phase in the catalyst under study is consistent with the observed high selectivity for benzene and indicates the predominant implementation of the dehydroxylation (DDO) pathway while maintaining the aromaticity of the ring.

The chemical state of cobalt in CoO/C can create an optimal balance between the reducing ability and the acid-base properties of the surface, which is necessary to activate the C–O bond without disturbing the aromaticity of the ring. This is consistent with the data of DFT calculations, according to which the interaction of oxygen atoms of phenolic compounds with Co surfaces leads to an elongation of C–O bonds, which serves as a molecular descriptor of their activation [34]. Although Co demonstrates accessible energy barriers for C_aryl_−O activation, when combined with a carbon support, it provides efficient bond cleavage without excessive product binding. At the same time, Fe and Ni, even at comparable or higher concentrations (for example, Ni—6.12% according to ICP-OES data), exhibit low activity, which indicates the key role of not only the quantitative but also the qualitative state of the active phase.

The high activity of the CoO/C catalyst may be due not only to the presence of CoO, but also to the specific structure of the carbon support containing both ordered graphite layers (graphite-2H) and defective areas contributing to the dispersion of the active phase and the transport of reagents. The absence of impurity oxide phases (for example, Co_3_O_4_ or spinels) indicates the purity of the synthesis and the stability of CoO under heat treatment conditions (700 °C).

Of interest is the difference in the reactivity of pyrocatechol and resorcinol. The increased yield of benzene from orthodiol may be due to the synergistic electronic effect of two neighboring hydroxyl groups contributing to the polarization and weakening of C–O bonds [25]. In the case of meta-substituted resorcinol, the spatial separation of the groups makes it difficult to activate together, and can also lead to competitive adsorption, which reduces the mobility of the molecule on the surface of the catalyst. These observations emphasize the importance of the geometric and electronic structure of the substrate in the design of catalytic systems for processing complex phenolic mixtures.

The results obtained confirm the prospects for the development of inexpensive, efficient and stable catalysts based on transition metals and a carbon substrate obtained by the activation of coke fines for the processing of phenolic compounds into valuable aromatic hydrocarbons.

## 4. Materials and Methods

### 4.1. Synthesis

The synthesis of catalysts for the catalytic processing of phenolic compounds was carried out by the method of wet impregnation of the support material (carbon sorbent and Al_2_O_3_) with solutions of salts of transition metals (Fe, Co, Ni).

The present work uses the same carbon sorbent sample that was synthesized and characterized in the study [23]. According to data from [23], the specific surface area of the material determined by the BET method is ~430 m^2^/g.

All catalysts were synthesized based on the theoretical metal content in the finished sample:

10 wt.% Fe for Fe_2_O_3_/C and Fe_2_O_3_–NiO/Al_2_O_3_;
5 wt.% Ni—for NiO/C and Fe_2_O_3_–NiO/Al_2_O_3_;
5 wt.% Co—for CoO/C and Fe_2_O_3_–CoO/C.

The actual metal content was confirmed by the ICP-OES method (Table 5). Small deviations may be associated with losses during washing, incomplete decomposition of precursors, or an analysis error.

A highly dispersed catalyst based on iron oxide on a carbon support (Fe_2_O_3_/C) was prepared by impregnating 9 g of carbon sorbent with a solution of 2.714 g of iron sulfate in 40 mL of distilled water. A mixture of carbon sorbent and iron sulfate solution was evaporated on a RE-201D rotary evaporator (Lanphan Company, Zhengzhou, China) for 60 min and, after water removal, was subjected to heat treatment in a quartz reactor without air access at a temperature of 500–560 °C. Thus, 9.86 g (94.6%) of Fe_2_O_3_/S was obtained. All remaining catalysts were prepared in the same way.

The CoO/C catalyst was obtained by impregnating 9 g of carbon sorbent with an aqueous solution of 4.76 g of cobalt sulfate heptahydrate (CoSO_4_·7H_2_O) in 40 mL of distilled water and, after removing the water, it was calcined in a quartz reactor without air access at a temperature of 700 °C. A total of 9.78 g (95.69%) was obtained.

The NiO/C catalyst was prepared by impregnation with a solution of 2.4 g of nickel sulfate (NiSO_4_·7H_2_O) in 40 mL of distilled water with 9.5 g of carbon sorbent at the rate of 5% Ni per carrier, then the water was removed using a rotary evaporator, and the remaining product was heat-treated in a quartz reactor without air access at a temperature of 800 °C. In this case, the crystallohydrate first loses crystallization water, then decomposes to form nickel oxide on a carbon substrate.

The binary catalyst Fe_2_O_3_–CoO/C was first prepared by impregnating 9 g of carbon sorbent with a solution of 2.714 g of ferrous sulfate in 40 mL of distilled water. After removal water and calcination at 560 °C, it was impregnated with a solution of 4.76 g of cobalt sulfate heptahydrate (CoSO_4_·7H_2_O) in 40 mL of distilled water and, after removing the water, calcined in a quartz reactor without air access at a temperature of 700 °C.

The Fe_2_O_3_–NiO/Al_2_O_3_ catalyst was prepared by impregnating 4.5 g of the support with a solution of 1.354 g of iron sulfate in 40 mL of distilled water, at the rate of 10% Fe per support. After removing the water using a rotary evaporator, the remaining product was heat-treated in a quartz reactor without air access at a temperature of 500–560 °C. As a result, iron sulfate decomposes to form iron oxide on an aluminum oxide substrate. A total of 4.97 g (99.4%) of Fe_2_O_3_/Al_2_O_3_ was obtained. Next, the obtained catalyst was dried and impregnated with a solution of 1.2 g of nickel sulfate (NiSO_4_·7H_2_O) in 40 mL of water and, after removing the water, calcined in a quartz reactor without air access at a temperature of 800 °C.

The CoO/Al_2_O_3_ catalyst was prepared by impregnating 9.5 g of Al_2_O_3_ with a solution of 2.42 g of cobalt sulfate (CoSO_4_·7H_2_O) in 40 mL of distilled water.

The NiO–CoO/Al_2_O_3_ catalyst was prepared by impregnating Al_2_O_3_ with a solution of 1.2 g of nickel sulfates (NiSO_4_·7H_2_O) and cobalt (CoSO_4_·7H_2_O) in 40 mL of distilled water with 9.5 g of Al_2_O_3_, then the water was removed using a rotary evaporator, and the remaining product was subjected to heat treatment in a quartz reactor without air access at a temperature of 850 °C.

### 4.2. Characteristics

The physicochemical properties of synthesized catalysts have also been studied using scanning electron microscopy and energy dispersion spectroscopy with elemental mapping (EDS mapping). The analysis was performed on a Zen 20 scanning electron microscope (Zeptools, Tongling, China) with a maximum resolution of 5 nm; maximum magnification, 360,000× *g*; accelerating voltage, 20 kV; energy resolution, eV Mn Ka 129 multichannel analyzer, number of channels 2048; reading speed, imp/sec 1,000,000; discreteness, eV/channel 10.

The phase composition of the synthesized catalysts was determined on a Rigaku smartLAB X-ray diffractometer (Rigaku Corporation, Tokyo, Japan) using CuKα-radiation (λ = 1.5406 Å) in the angle range (2θ) 10–100° and increments of 0.01°. Diffractograms were processed to identify compounds using the PDF 2 database.

Quantitative analysis of inductively coupled plasma optical emission spectrometry (ICP-OES) to determine the element content in synthesized catalysts was performed on a Spectro Arcos special photometer (Spectro TC Group of Companies, Moscow, Russia) with an operating spectral range from 130 to 770 nm and an optical resolution of 8.5 pm in the range from 130 to 340 nm and a focal length of 750 mm.

Hydrogenation of phenol, pyrocatechol and resorcinol was performed in a 50 mL CJF-0.05 autoclave (Zhengzhou Keda Machinery and Instrument, Zhengzhou, China).

For hydrogenation, 1 g of the studied organic compound and 0.1 g of the obtained catalyst were taken.

All experiments on the hydrogenation of phenol in the presence of obtained carbon sorbent and various catalysts (Fe_2_O_3_/C, CoO/C, NiO/C, CoO/Al_2_O_3_, Fe_2_O_3_–CoO/C, Fe_2_O_3_–NiO/Al_2_O_3_, NiO–CοO/Al_2_O_3_) and catechol in the presence of a catalyst CoO/C were carried out under identical conditions: temperature of 420 °C, a hydrogen pressure of 6.5 MPa, the reaction duration of 120 min.

Hydrogenation of resorcinol in the presence of a CoO/C catalyst was carried out at a temperature of 420 °C, a hydrogen pressure of 6 MPa, and a reaction duration of 120 min.

The reactions were carried out under solvent-free conditions, where the phenolic compound itself served as the liquid phase at the reaction temperature. After the reaction was completed, the autoclave was cooled to room temperature before releasing the hydrogen pressure. This ensured the condensation of all volatile organic products (including benzene) back into the liquid phase.

The analysis of the hydrogenation products of phenol, pyrocatechol and resorcinol in the presence of synthesized catalysts was carried out using gas–liquid chromatography (GLC) on a Crystallux 4000 M chromatograph (NPF Meta-Chrom, Yoshkar-Ola, Russia) with a FID detector module on a 30 m ZB-5 column × 0.32 mm × 0.25 microns.

The concentrations of the components in the reaction mixture (% by weight) were determined by gas–liquid chromatography using normalization of the areas of all registered organic peaks.

The conversion of phenol (%) was calculated using the formula:

X = 100% − C_phenol_, (1)

where X is the conversion (%), and C_phenol_ is the mass fraction of unreacted phenol in the reaction products (%).

Since no other organic products other than phenol and benzene were found in the chromatograms, the yield of benzene (%) was assumed to be equal to its mass fraction in the reaction mixture, which, in the absence of by-products, coincides with the conversion of phenol.

For example, when using the CoO/C catalyst, the residual phenol content was 37.64% (Table 1); therefore, the phenol conversion and benzene yield were 62.36%.

The catalytic stability and the reusability of the CoO/C catalyst were evaluated over 5 consecutive cycles in the phenol hydrogenation reaction. After each cycle, the CoO/C catalyst along with the hydrogenation products were discharged from the reactor. The reactor was washed with isopropyl alcohol. The CoO/C catalyst was washed with isopropyl alcohol 3 times and dried at 90 °C for 60 min. After that, the catalyst, washed and dried CoO/C catalyst and a new batch of phenol were loaded back into the reactor for the next cycle.

## 5. Conclusions

The observed differences in the reactivity of ortho- and meta-isomers of phenolic compounds emphasize the crucial role of the spatial distribution of hydroxyl groups in the substrate molecule and their effect on the mechanism of dehydroxylation. The increased yield of benzene from pyrocatechol (orthodiol) compared with resorcinol (meta-diol) indicates that the adjacent arrangement of functional groups contributes to the weakening of C–O bonds and stabilization of the transition state on the catalyst surface, while the meta-configuration may complicate complete dehydroxylation due to steric and electronic restrictions.

The results obtained demonstrate the high prospects of using low-cost cobalt-containing catalysts on a carbon support obtained from coke fines from the Shubarkol field for the selective processing of phenolic compounds into valuable aromatic hydrocarbons, primarily benzene. The CoO/C catalyst showed not only high activity and selectivity, but also stability over five consecutive catalytic cycles. The selectivity of the CoO/C catalyst was evaluated by the yield of benzene, which is the target product of the dehydroxylation of phenol, pyrocatechol, and resorcinol. No by-products (cyclohexanol, cyclohexane, dimers, etc.) were found in the chromatograms, which indicates a high selectivity (>99%) specifically for benzene. It was found that when using the CoO/C catalyst, the maximum benzene yield was 62.36% when hydrogenating phenol, 63.16% when hydrogenating pyrocatechol, and 48.64% when hydrogenating resorcinol.

At the same time, the binary compositions studied—Fe–Co/C and Fe_2_O_3_–NiO/Al_2_O_3_—demonstrated significantly lower catalytic activity and did not reveal a synergistic effect between the components. This allows us to conclude that it is inappropriate to use these binary systems in their current configuration and highlights the advantage of a cobalt-based monoxide catalyst.

Thus, the proposed approach combines environmental rationality (utilization of industrial waste—coke fines and phenolic compounds) with economic efficiency (abandonment of expensive precious metals), paving the way for the development of sustainable technologies for the deep processing of secondary resources into valuable chemical products.

## Figures and Tables

**Table 1:** Results of phenol hydrogenation in the presence of carbon sorbent without deposited metal.

Component in Hydrogenation Products	Concentration of Component in Hydrogenation Products, (%)
Benzene	3.7
Phenol	96.3

**Table 2:** Results of inductively coupled plasma optical emission spectrometry (ICP-OES) analysis.

No.	Elements	Elements of Sample Number and Content (Mass %)						
		1	2	3	4	5	6	7
		Fe_2_O_3_/C	CoO/C	NiO/C	CoO/Al_2_O_3_	Fe_2_O_3_–CoO/C	Fe_2_O_3_–NiO/Al_2_O_3_	NiO–CoO/Al_2_O_3_
1	Al	1.182	1.144	1.404	>50	1.184	>50	>50
2	Fe	8.598	0.797	0.916	0.711	4.380	3.273	1.643
3	Co	0.0139	4.4119	0.0504	5.303	4.8349	0.0212	4.621
4	Ni	0.0060	0.0190	6.1210	0.0127	0.0100	5.4620	5.184
5	Cr	0.0022	0.0013	0.0011	<0.0010	0.0125	<0.0010	<0.0010

**Table 3:** Results of phenol hydrogenation in the presence of the obtained catalysts.

A Component in Hydrogenation Products	Catalyst						
	Fe_2_O_3_/C	CoO/C	NiO/C	CoO/Al_2_O_3_	Fe_2_O_3_–CoO/C	Fe_2_O_3_–NiO/Al_2_O_3_	NiO–CoO/Al_2_O_3_
	Component Concentration in Products (%)						
Benzene (%)	10.53	62.36	18.17	6.5	10.98	12.53	9.65
Phenol (%)	89.17	37.64	81.04	92.03	87.83	83.53	90.35

**Table 4:** Results of phenol hydrogenation in the presence of the prepared CoO/C catalyst over five cycles.

Component Concentration in Hydrogenation Products	Cycle Number				
	1 (a)	2 (b)	3 (c)	4 (d)	5 (e)
Benzene (%)	62.36	59.19	57.02	31.95	27.35
Phenol (%)	37.64	40.81	42.98	68.05	72.65

**Table 5:** Hydrogenation products of pyrocatechol and resorcinol in the presence of a CoO/C catalyst.

A Component in Hydrogenation Products	A Compound That Undergoes Hydrogenation	
	Pyrocatechol	Resorcinol
	Component Concentration in Hydrogenation Products (%)	
Benzene	63.16	48.64
Phenol	36.84	45.82
1,3-Dihydroxybenzene (resorcinol)	-	5.54

**Table 6:** Interpretation of the total spectrum of the element content in the resulting carbon sorbent, which is used as a catalyst substrate.

Element	Line Type	Weight, %	Sigma Weight, %	Atom., %
C	K-serie	86.56	0.22	91.44
O	K-serie	8.36	0.2	6.63
Na	K-serie	0.1	0.03	0.05
Mg	K-serie	0.09	0.02	0.05
Al	K-serie	0.9	0.03	0.42
Si	K-serie	1.52	0.04	0.69
S	K-serie	0.35	0.03	0.14
Cl	K-serie	0.43	0.03	0.15
Ca	K-serie	0.41	0.04	0.13
Fe	K-serie	1.29	0.1	0.29
Total		100		100

**Table 7:** Interpretation of the total spectrum of the element content in the obtained CoO/C catalyst.

Element	Line Type	Weight, %	Sigma Weight, %	Atom., %
C	K-serie	67.75	0.31	85.56
O	K-serie	7.57	0.18	7.18
Al	K-serie	0.46	0.03	0.26
Si	K-serie	0.69	0.03	0.37
S	K-serie	2.34	0.05	1.11
Cl	K-serie	0.13	0.03	0.06
Ca	K-serie	0.29	0.04	0.11
Fe	K-serie	0.81	0.09	0.22
Co	K-serie	19.96	0.26	5.14
Total		100		100

**Table 8:** Comparative table of optimal conditions for the catalytic treatment of phenols and benzene yields using the catalysts obtained in this work and in the literature data.

Catalyst	Substrate	Catalytic Treatment Conditions	Benzene Concentration in Products (%)
La_0.9_Ni_0.5_Co_0.5_O_3_-δ [32]	Phenol	T = 350 °C; P (H_2_) = 0.1 MPa	32.4
Ni_2_P/SiO_2_ [33]	Anisole + phenol	T = 400 °C; P (H_2_) = 0.5 MPa	95.7
CoO/C	Phenol	T = 420 °C; P (H_2_) = 6.5 MPa	62.36

## Data Availability

The original contributions presented in this study are included in the article. Further inquiries can be directed to the corresponding author.

## References

[1] Zheng M., Bai Y., Han H., Zhang Z., Xu C., Ma W., Ma W. (2021). Robust removal of phenolic compounds from coal pyrolysis wastewater using anoxic carbon-based fluidized bed reactor. J. Clean. Prod..

[2] Khan M.J., Wibowo A., Karim Z., Posoknistakul P., Matsagar B.M., Wu K.C.-W., Sakdaronnarong C. (2024). Wastewater Treatment Using Membrane Bioreactor Technologies: Removal of Phenolic Contaminants from Oil and Coal Refineries and Pharmaceutical Industries. Polymers.

[3] Dong S., Feng G. (2025). A Comprehensive Review of Catalytic Hydrodeoxygenation of Lignin-Derived Phenolics to Aromatics. Molecules.

[4] Nayak R.R., Gupta N.K. (2025). Renewable aromatic production from waste: Exploring pathways, source materials, and catalysts. Green Chem..

[5] Busca G. (2021). Production of Gasolines and Monocyclic Aromatic Hydrocarbons: From Fossil Raw Materials to Green Processes. Energies.

[6] Lang M., Li H. (2022). Toward Value-Added Arenes from Lignin-Derived Phenolic Compounds via Catalytic Hydrodeoxygenation. ACS Sustain. Chem. Eng..

[7] Gundekari S., Karmee S.K. (2020). Selective Synthesis of Cyclohexanol Intermediates from Lignin-Based Phenolics and Diaryl Ethers Using Hydrogen over Supported Metal Catalysts: A Critical Review. Catal. Surv. Asia.

[8] Wang X., Arai M., Wu Q., Zhang C., Zhao F. (2020). Hydrodeoxygenation of lignin-derived phenolics—A review on the active sites of supported metal catalysts. Green Chem..

[9] Lu X., Guo H., Wang D., Xiu P., Qin Y., Chen J., Xu C., Gu X. (2021). A review on catalytic conversion of lignin into high-value chemicals over Ni-based catalysts. Biomass Convers. Biorefin..

[10] Zhang C., Zhang X., Wu J., Zhu L., Wang S. (2022). Hydrodeoxygenation of lignin-derived phenolics to cycloalkanes over Ni–Co alloy coupled with oxophilic NbOx. Appl. Energy.

[11] Ding S., Zhu X.L. (2024). Tuning Strong Metal-Support Interactions for Enhancing Direct Deoxygenation of Biomass-Lignin Derived Phenolics. ChemCatChem.

[12] Fang H., Zheng J., Luo X., Du J., Roldan A., Leoni S., Yuan Y. (2017). Product tunable behavior of carbon nanotubes-supported Ni–Fe catalysts for guaiacol hydrodeoxygenation. Appl. Catal. A Gen..

[13] Han Q., Rehman M.U., Wang J., Rykov A., Gutiérrez O.Y., Zhao Y., Wang S., Ma X., Lercher J.A. (2019). The synergistic effect between Ni sites and Ni–Fe alloy sites on hydrodeoxygenation of lignin-derived phenols. Appl. Catal. B Environ..

[14] Wei Z., Li Y., Wang J., Li H., Wang Y. (2018). Chemoselective hydrogenation of phenol to cyclohexanol using heterogenized cobalt oxide catalysts. Chin. Chem. Lett..

[15] Tavizón-Pozos J.A., Suárez-Toriello V.A., del Ángel P., de los Reyes J.A. (2016). Hydrodeoxygenation of phenol over sulfided CoMo catalysts supported on a mixed Al_2_O_3_–TiO_2_ oxide. Int. J. Chem. React. Eng..

[16] Cao W., Li Z., Zhang Y., Fu P. (2024). Hydrodeoxygenation of Lignin Phenol Derivatives to Aromatic Hydrocarbons: A Mini-Review of Metal/Acid Bifunctional Catalysts. Energy Fuels.

[17] Taghvaei H., Moaddeli A., Khalafi-Nezhad A., Iulianelli A. (2021). Catalytic hydrodeoxygenation of lignin pyrolytic-oil over Ni catalysts supported on spherical Al-MCM-41 nanoparticles: Effect of Si/Al ratio and Ni loading. Fuel.

[18] Li S., Guo L., He X., Qiao C., Tian Y. (2022). Synthesis of uniform Ni nanoparticles encapsulated in ZSM–5 for selective hydrodeoxygenation of phenolics. Renew. Energy.

[19] Yaghi A., Ali L., Shittu T., Kuttiyathil M.S., Khaleel A., Altarawneh M. (2024). Hydrodeoxygenation of Vapor Anisole over Nickel/Cobalt and Alumina/Zeolite Supported Catalysts. Catal. Surv. Asia.

[20] Hill J.M. (2017). Sustainable and/or waste sources for catalysts: Porous carbon development and gasification. Catal. Today.

[21] Kurniawansyah F., Pertiwi R.D., Perdana M., Al-Muttaqii M., Roesyadi A. (2020). Development of bamboo-derived activated carbon as catalyst support for glucose hydrogenation. Mater. Sci. Forum.

[22] Villora-Picó J.J., González-Arias J., Baena-Moreno F.M., Reina T.R. (2024). Renewable Carbonaceous Materials from Biomass in Catalytic Processes: A Review. Materials.

[23] Ordabaeva A.T., Muldakhmetov Z.M., Meiramov M.G., Kim S.V. (2025). Activation of Coke Fines Using CO_2_ and Steam: Optimization and Characterization of Carbon Sorbents. Molecules.

[24] Hadi Abdullahi B., Muhammad A.U. (2025). Activated carbon-supported catalysts for oil-to-hydrocarbon conversion: A review. Acad. Green Energy.

[25] Pan Z., Puente-Urbina A., Bodi A., van Bokhoven J.A., Hemberger P. (2021). Isomer-Dependent Catalytic Pyrolysis Mechanism of the Lignin Model Compounds Catechol, Resorcinol and Hydroquinone. Chem. Sci..

[26] Zhou J., An W. (2020). Unravelling the Role of Oxophilic Metal in Promoting Deoxygenation of Catechol on Ni-Based Alloy Catalyst. Catal. Sci. Technol..

[27] Kirkwood K., Jackson S.D. (2021). Competitive Hydrogenation and Hydrodeoxygenation of Oxygen-Substituted Aromatics over Rh/Silica: Catechol, Resorcinol and Hydroquinone. Top. Catal..

[28] Song W., Liu Y., Baráth E., Zhao C., Lercher J.A. (2015). Synergistic Effects of Ni and Acid Sites for Hydrogenation and C–O Bond Cleavage of Substituted Phenols. Green Chem..

[29] Lonchay W., Bagnato G., Sanna A. (2022). Highly selective hydropyrolysis of lignin waste to benzene, toluene and xylene in presence of zirconia supported iron catalyst. Bioresour. Technol..

[30] Hillerová E., Vít Z., Zdražil M., Shkuropat S.A., Bogdanets E.N., Startsev A.N. (1990). Comparison of Carbon- and Alumina-Supported Nickel–Molybdenum Sulphide Catalysts in Parallel Hydrodenitrogenation and Hydrodesulphurisation. Appl. Catal..

[31] Jacobs G., Das T.K., Zhang Y., Li J., Racoillet G., Davis B.H. (2002). Fischer–Tropsch Synthesis: Support, Loading, and Promoter Effects on the Reducibility of Cobalt Catalysts. Appl. Catal. A Gen..

[32] Peng L., Jiang W., Li N., Zhong X., Lian C., Li Z. (2025). Three-Dimensionally Ordered Macroporous La_0.9_Ni_0.5_Co_0.5_O_3-δ_ Perovskite Catalyst with Enhanced Catalytic Performance for Phenol Hydrodeoxygenation. J. Solid State Chem..

[33] Li Y., Fu J., Chen B. (2017). Highly Selective Hydrodeoxygenation of Anisole, Phenol and Guaiacol to Benzene over Nickel Phosphide. RSC Adv..

[34] Morteo-Flores F., Roldan A. (2022). Mechanisms and Trends of Guaiacol Hydrodeoxygenation on Transition Metal Catalysts. Front. Catal..

